# Realtime detection of spontaneous circulation in humans during cardiopulmonary resuscitation using a continuous hands-free carotid Doppler: a pilot study

**DOI:** 10.1016/j.resplu.2025.101080

**Published:** 2025-09-03

**Authors:** Guro Mæhlum Krüger, Sunniva Gjerald Birkeli, Øystein Myrlund Hansen, Bjørn Ove Faldaas, Anders Norvik, Hedda Juni Lund, Gregory Louis Egil Hautois, John Helge Flage, Jon Urteaga, Torbjørn Hergum, Hans Torp, Eirik Skogvoll, Charlotte Björk Ingul

**Affiliations:** a Clinic of Anesthesia and Intensive Care, St. Olavs Hospital, Trondheim University Hospital, Trondheim, Norway; b Department of Circulation and Clinical Imaging, Norwegian University of Science and Technology, Trondheim, Norway; c Faculty of Nursing and Health Science, Nord University, Bodø, Norway; d Department of Emergency Medicine, Nordland Hospital Trust, Bodø, Norway; e University of the Basque Country, Spain; f Cimon Medical AS, Trondheim, Norway

**Keywords:** Advanced life support (ALS), Cardiopulmonary resuscitation (CPR), Pulse check, Return of spontaneous circulation (ROSC), Spontaneous circulation, Chest compressions, Carotid Doppler ultrasound, Wearable Doppler ultrasound, Operator independent Doppler ultrasound, Mechanical chest compression

## Abstract

**Background:**

The resuscitation society calls for precision-guided cardiopulmonary resuscitation (CPR), as current methods are inaccurate and time-consuming. RescueDoppler, a novel hands-free Doppler ultrasound system, continuously measures carotid blood flow during CPR. This pilot study assessed its performance, safety, and ability to detect chest compression-generated blood flow, spontaneous circulation, and return of spontaneous circulation (ROSC).

**Method:**

We investigated RescueDoppler in adult cardiac arrest patients at two centres, in-hospital (IHCA) and out-of-hospital (OHCA). The cardiac arrest team placed the RescueDoppler probe over the left common carotid artery with a self-adhesive patch, collecting blinded data during CPR. Data were later interpreted and time-synchronized with defibrillator data using custom MATLAB® software.

**Results:**

RescueDoppler was used in 26 IHCA and 36 OHCA patients from October 2023 to September 2024. Carotid blood flow curves were analyzed in 36 patients and synchronized with defibrillator data in 30. The RescueDoppler identified blood flow velocities generated by chest compressions and detected spontaneous circulation during rhythm checks. ROSC was defined by the presence of systolic and diastolic blood flow. No adverse events were reported but there were 22 device deficiencies mostly related to the self-adhesive patch and connecting cable. The system is user-friendly and requires minimal training.

**Conclusion:**

Real-time detection of blood flow in the carotid artery with hands-free Doppler ultrasound during CPR is safe and feasible, although the fastening patch and host unit need optimization. The RescueDoppler system detects spontaneous circulation and ROSC during rhythm checks and ongoing chest compressions. Further research is required to confirm clinical relevance.

## Introduction

Successful outcome after cardiac arrest relies on the restoration of heart activity and the resumption of adequate blood flow and oxygen delivery to vital organs. High-quality cardiopulmonary resuscitation (CPR) includes effective chest-compressions and early shock when indicated.^1^, ^2^ However, assessing CPR quality remains challenging.^1^ With some exceptions (e.g., in children and pregnant women) current advanced life support (ALS) algorithms follow a “one-size-fits-all” standardized approach, disregarding individual anatomy and physiologic response.^1^, ^2^ Recognizing these limitations, further research into areas such as the optimal chest compression site and depth, when to resume compressions after defibrillation, and individualized medication dosing is of great interest.^3^, ^4^

The common carotid artery is well suited for direct non-invasive measurement of blood flow during CPR and is easily accessible with known anatomic landmarks. The use of point-of-care ultrasound to evaluate blood flow in the carotid or femoral arteries during CPR has increased during the last decade.^5^, ^6^, ^7^ However, this method has limitations as it might cause additional or prolonged interruptions of chest compressions, requires a skilled operator, and does not provide continuous hemodynamic information.^8^, ^9^, ^10^ Considering that most cardiac arrests happen in a resource limited out-of-hospital context^11^; an easy-to-use, non-invasive continuous monitoring of the circulation would be of great interest. Hence, we developed a novel hands-free ultrasound Doppler-system, RescueDoppler, to continuously measure blood flow in the common carotid artery.^12^

The RescueDoppler system has been successfully tested in animals and can reliably detect blood flow during low blood pressures, identify return of spontaneous circulation (ROSC) during cardiac arrest, differentiate between true- and pseudo-pulseless electrical activity, evaluate efficiency of chest compressions, and identify spontaneous blood flow during ongoing chest compressions.^12^, ^13^, ^14^

This study aimed to evaluate the feasibility and safety of the RescueDoppler system to detect carotid blood flow as generated by chest compressions or spontaneous circulation, to possibly replace manual pulse palpation in patients with cardiac arrest.

## Materials and methods

### Study design and settings

The study involved adult patients with in- and out-of-hospital cardiac arrest (IHCA, OHCA) in urban areas of mid- and northern Norway; at St. Olav's Hospital, Trondheim University Hospital (IHCA and OHCA) and Nordland Hospital Trust, Bodø (OHCA). The study personnel included all physicians, physician- staffed helicopter emergency medical service crew members, paramedics, nurse anesthetists, and anaesthesiologists involved in cardiac arrest resuscitation on study sites; all received protocol-specific instruction. After every patient inclusion, a questionnaire regarding the incident and use of equipment was completed by study personnel (Appendix 2), followed by an unstructured interview by the study group.

### The RescueDoppler prototype system

**Fig. 1:**
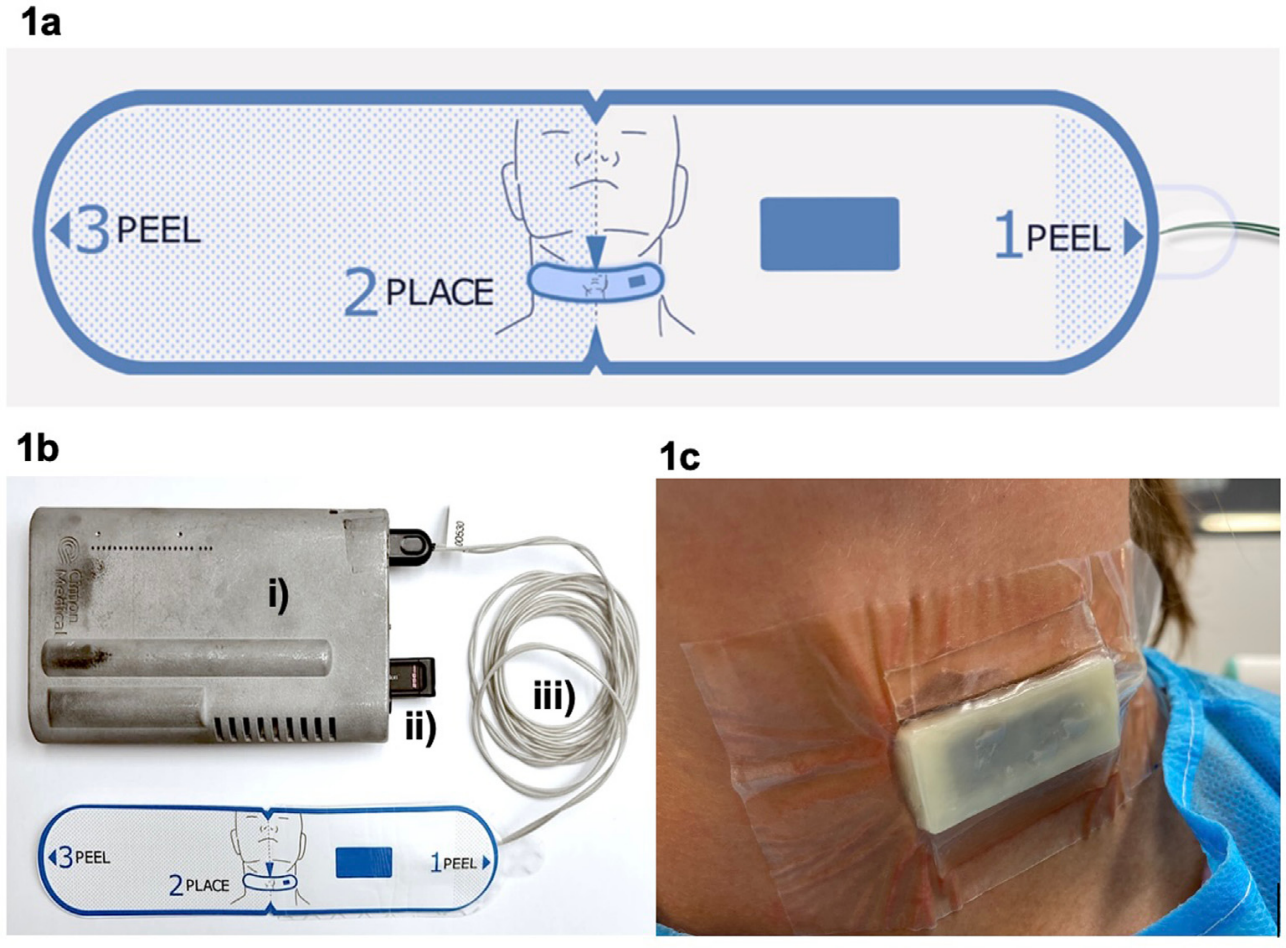
(a) The RescueDoppler application guide. (b) The RescueDoppler system: (i) host unit with ultrasound scanner and computer, (ii) USB drive, (iii) extension cable. (c) The RescueDoppler probe and self-adhesive patch are shown in place, following the removal of the application guide.

The RescueDoppler prototype system developed by Cimon Medical and the Norwegian University of Science and Technology (NTNU) includes a carotid Doppler probe with two transducer elements tilted ±30°. RescueDoppler utilizes pulsed-wave Doppler technology, enabling its transducer elements to both transmit and receive ultrasound signals. The transducer elements are unfocused, measure 30 mm in length, and have a central frequency of 4 MHz. Data from the probe are processed by the host unit (Fig. 1b) which integrates an ultrasound scanner, a battery, and a computer with signal analysis software. The RescueDoppler probe is attached to the skin by a self-adhesive patch and an application guide (Fig. 1a, c).

Ultrasound echoes are received and sampled at multiple depths from the skin surface, providing Doppler measurements of blood flow velocities from the carotid artery and the jugular vein. Changes in blood flow are displayed in real-time as a Doppler spectrogram, in combination with automatic waveform analysis that provides numerical values for systolic, diastolic, and mean velocities. Tilting of the probe introduces uncertainty in blood flow velocities, as the exact insonation angle remains unknown. As a result, interpretation was based on blood flow patterns and velocity trends rather than absolute values. The technology is user-independent, and the unfocused ultrasound beam allows monitoring of the subject for up to several hours due to low acoustic output compared to standard Doppler devices. In this pilot study the system was used as a “black box” and did not show any diagnostic information. Data collected from the system were recorded for off-line, retrospective analysis. The depth or focus area of the ultrasound signals are manually selected by the investigators.

The RescueDoppler system was improved several times during the study. Detailed information can be found in Supplementary text (Appendix 1). The application guide is patented (International (PCT) Patent Application No. PCT/EP2024/058678).

### Defibrillators

Ambulances at Nordland Health Trust Bodø and in Trondheim all used CORPULS 3 defibrillators (software 4.2.2). Data were extracted via a memory card and uploaded to a secure database.

At St. Olav́s Hospital, Trondheim University Hospital, ZOLL R-series defibrillator (software 19.03) was used, with data initially extracted manually and later automatically via a secure WiFi connection. CPR feedback devices (CorPatch, Zoll CPR-D-padz) and EtCO_2_ measurement were available for both the pre- and in-hospital teams.

### Patients

Adult patients aged 18 years and older suffering cardiac arrest and receiving ALS between September 2023 and September 2024 were considered for inclusion. Patients were excluded if resuscitation was not continued after RescueDoppler application, halted due to a do-not-resuscitate order, or if extensive injuries prevented patch application.

Recordings were excluded from further data analysis if they did not meet study criteria or contained missing/uninterpretable data. The investigators reviewed all patients considered for inclusion.

### Conduct of the study

After ensuring ALS per national CPR guidelines,^15^ study personnel placed the RescueDoppler probe with the self-adhesive patch over the left common carotid artery by means of the application guide (Fig. 1). The RescueDoppler continuously collected data to the host unit for later analysis, leaving the study personnel blinded to the flow velocity curves. ECG, chest compression accelerometer, and EtCO_2_ data if available were also collected.

### Data collection and management

Demographic and cardiac arrest data were collected using the Utstein template, a RescueDoppler questionnaire (Supplementary material, Appendix 2), and unstructured interviews with study personnel if needed. Medical history and medication information were collected from electronic records after consent. Outcomes were assessed from medical records 30 days post-arrest.

RescueDoppler data were recorded, converted and analyzed using a custom-made MATLAB® (R2023b) software, in most cases time-synchronized with defibrillator data and visually evaluated by the investigator (GMK).

### Statistical analysis

Based on incidence figures^11^, we expected about 90 in-hospital and 40 out-of-hospital episodes for inclusion during the planned study period.

RescueDoppler data were manually assessed by the investigator (GMK), with secondary evaluation by the principal investigator (CBI) to ensure dataset integrity. Data were analyzed using descriptive statistics, with mean and standard deviations for continuous and normally distributed data, and median with interquartile range (IQR) for non-normal data.

### Safety, legal and ethical aspects

A clinical study monitor (Research Department, St. Olav́s Hospital, Trondheim University Hospital) regularly visited study sites per the monitoring plan. All safety concerns, device deficiencies or adverse events were reported and handled by the project group.

The study followed ICH GCP; ISO 14155:2020) and the Declaration of Helsinki guidelines. It was approved by The National Ethical Committee for investigational devices (REK KULMU 582681) and the Norwegian Medical Products Agency (CIV-NO-23-05-043033) with a Data Protection Impact Assessment (DPIA 220374). The study is registered in Clinical trials (Identifier: NCT06599073).

Informed consent was obtained post- cardiac arrest by legal representative or relatives if patients were unable to provide consent themselves.

## Results

### Demographic and cardiac arrest characteristics

Inclusion rates were 27 % for in-hospital events and 64 % for out- of- hospital events. Reasons for non-inclusion included ROSC before the emergency team‘s arrival (*n* = 32), do-not-resuscitate orders (*n* = 16), RescueDoppler equipment unavailable (*n* = 21), forgetting to apply the RescueDoppler equipment (*n* = 15), location in intensive care units without emergency team alerts (*n* = 2), left side of the neck occupied (*n* = 2), and other reasons (*n* = 2).

**Fig. 2:**
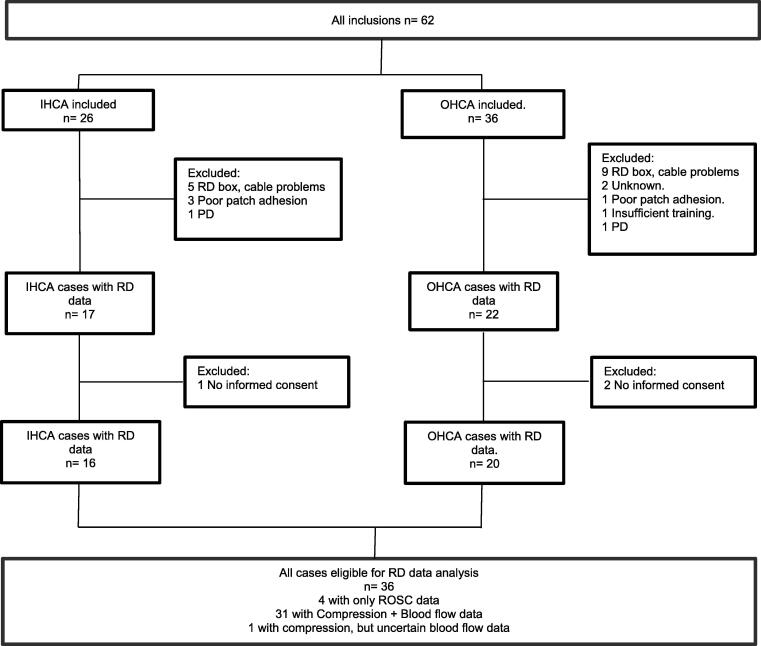
A total of 62 patients were included in the study (26 in-hospital and 36 out-of-hospital). RescueDoppler data were available for 17 in- hospital cardiac arrest and 22 out-of-hospital cardiac arrest patients. Informed consent was withdrawn by next of kin for 3 patients, resulting in a final cohort of 36 patients for analysis of demographics, cardiac arrest characteristics and RescueDoppler data. RD = RescueDoppler, OHCA = out of-hospital cardiac arrest, IHCA = in-hospital cardiac arrest, PD = Protocol Deviation.

In total, sixty-two patients were included, 26 IHCA and 36 OHCA (Fig. 2). RescueDoppler data were available for 39 patients (63 %), among these 36 were eligible for further data analysis (58 %). Demographics and descriptive cardiac arrest data are presented in Table 1.

**Table 1:** Demographics, initial rhythm, causes of CA and information about ROSC.

Inclusions with RescueDoppler data, *n* (%)	36 (100)
Men, *n* (%)	27 (75)

Age (years), mean (range)	
Women	65 (35–79)
Men	65 (15–86)

Location *n* (%)	
IHCA	16 (44.4)
OHCA	20 (55.6)

First observed rhythm, *n* (%)	
PEA	16 (44.4)
Asystole	15 (41.7)
VF	5 (13.9)

Cause of cardiac arrest, *n* (%)	
Cardiac	10 (27.8)
Unknown	6 (16.7)
Pulmonary	6 (16.7)
Trauma	3 (8.3)
Strangulation	2 (5.6)
Sepsis	2 (5.6)
Hypovolemia	2 (5.6)
Intoxication	2 (5.6)
Other	2 (5.6)
Missing data	1 (2.8)

Prior carotid artery disease, *n* (%)	0 (0)

ROSC, *n* (%)	
ROSC (IHCA and OHCA)	19 (52.8)
OHCA	7 (19.4)
IHCA	12(33.3)

CA = Cardiac arrest IHCA = In-hospital cardiac arrest OHCA = out-of hospital cardiac arrest PEA = pulseless electric activity VF = ventricular fibrillation.

The mean body mass index (BMI, kg/m^2^) was 26.1 (range 19.8–35.9) in women and 28.9 (range 16.6–41.0) in men. A mechanical compression device (LUCAS2®) was used in four IHCA and in four OHCA patients. A chest compression accelerometer was employed in 15 patients and 27 patients had EtCO_2_ monitoring. Intravenous Adrenaline was administered in 31 patients and Amiodarone bolus in 6 patients. Three patients received mechanical circulatory support (extracorporeal membrane oxygenation, Impella®, or intra-aortic balloon pump) following return of spontaneous circulation.

Nineteen patients (53 %) achieved ROSC after which 14 subsequently died. Overall, 31 (86 %) patients died within 30 days.

### Feasibility of the RescueDoppler system

The median time from cardiac arrest recognition to RescueDoppler patch application was 6 min 30 s (IQR 3 min 45 s–12 min). The patch remained connected for a mean duration of 37 min (range 9–90 min).

Device deficiencies were reported in 22 of the 62 included patients. Some of the device deficiencies were multiple: 15 related to poor patch adhesion; six related to the host unit; 10 to cable connection issues; four to charging of the host unit, and four due to full memory (Supplementary material, Appendix 3).

### RescueDoppler blood flow data

RescueDoppler blood flow velocity curves were identified in 35 of 36 (97 %) analyzed patients. The mean carotid artery depth was 19.9 mm (range 10–35) in women and 20.0 mm (range 12–35) in men as measured by color M-mode. Venous and arterial blood flow were reliably differentiated by color M-mode in 29 (80 %) cases. Venous signal visualization failed in one case, and the distinction was uncertain in six cases. Time synchronization and annotation of RescueDoppler curves with defibrillator data were completed in 83 %. Defibrillator data were missing for six patients.

**Fig. 3:**
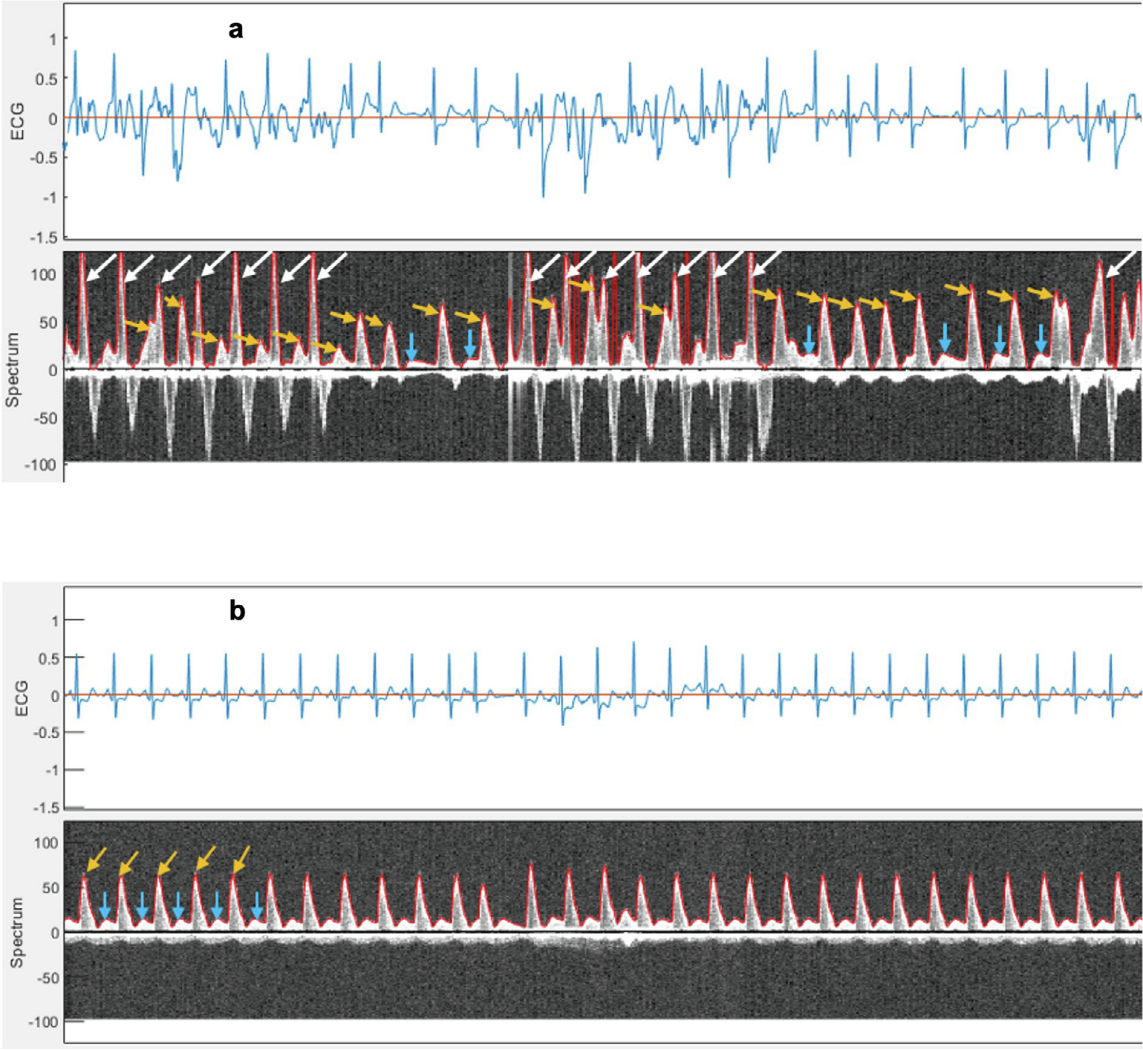
(a) Spontaneous circulation observed during rhythm assessments in the intervals between chest compressions. The upper panel displays the electrocardiogram (ECG), the lower panel shows carotid blood flow velocity. (b) Return of spontaneous circulation. Upper panel electrocardiogram (ECG) with sinus rhythm and lower panel carotid blood flow velocity, showing both systolic and diastolic velocities. White arrows indicate carotid blood flow generated by chest compressions. Yellow arrows represent carotid blood flow during spontaneous circulation. Blue arrows show carotid diastolic blood flow. In the Doppler spectrum, positive velocities (above the baseline) signify blood flow directed towards the brain. (For interpretation of the references to color in this figure legend, the reader is referred to the web version of this article.)

**Fig. 4:**
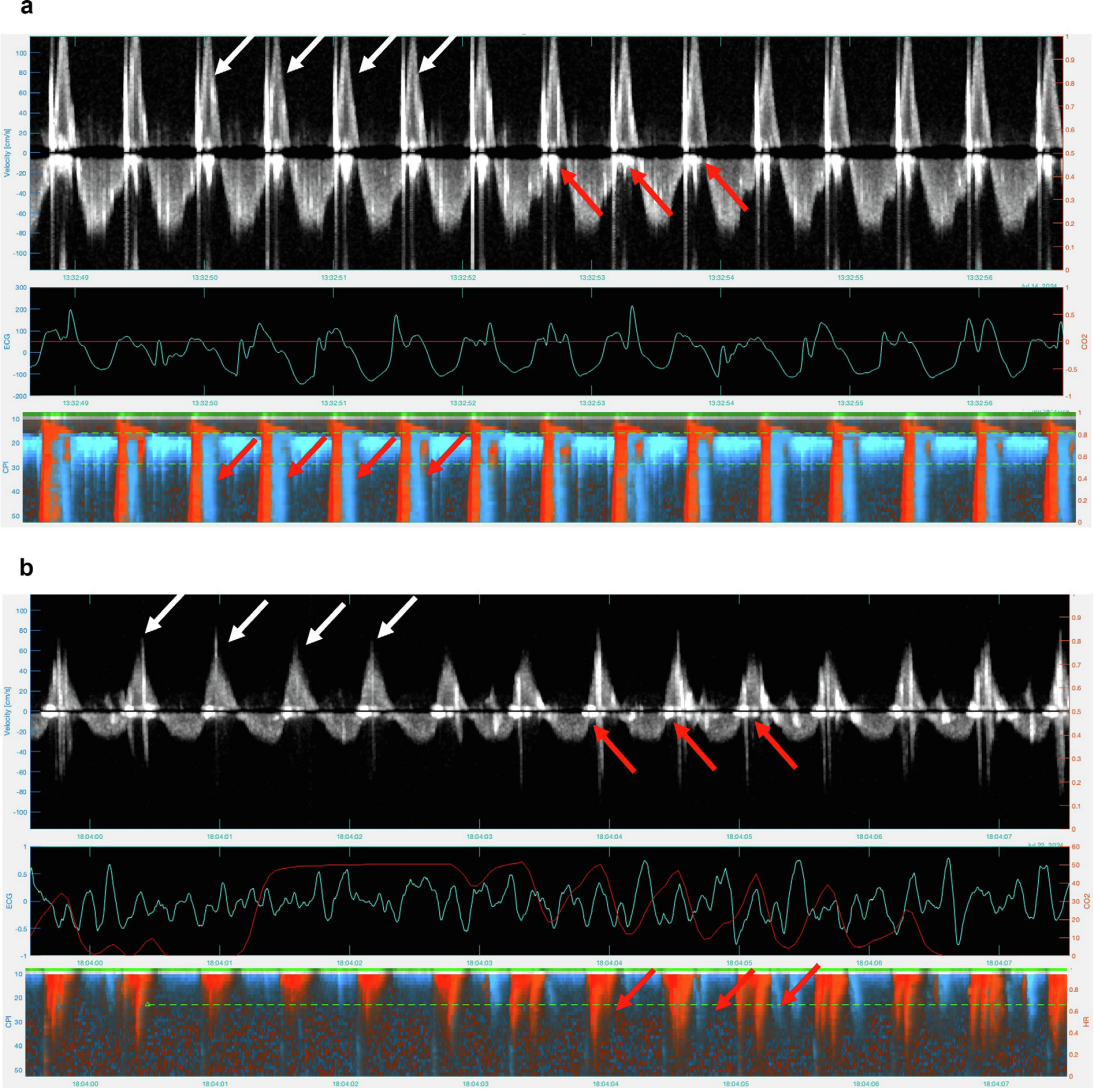
Blood flow generated by chest compressions. Upper panel shows blood flow velocity, middle panel shows electrocardiogram (ECG, blue curve) and end tidal carbon dioxide (EtCO_2_, red curve), lower panel shows color Doppler M-mode. Positive velocities of the Doppler spectrum pointing upwards (above baseline) identifies flow towards the brain. White arrows indicate peak systolic blood flow velocities, red arrows mark artifacts caused by chest compressions. (a) Mechanical chest compression (LUCAS2®) generated blood flow velocities. Positive carotid blood flow at 120 cm/sec (white arrows). The red arrows highlight artifacts caused by chest compressions visible around the baseline in upper panel, with corresponding artifacts also present in the lower panel. (b) Manual chest compression generated blood flow velocities. Positive carotid blood flow at 60 cm/sec (white arrows). The red arrows indicate white artifacts from chest compressions around baseline in upper panel and corresponding intense red and blue artifacts in the lower panel. (For interpretation of the references to color in this figure legend, the reader is referred to the web version of this article.)

**Fig. 5:**
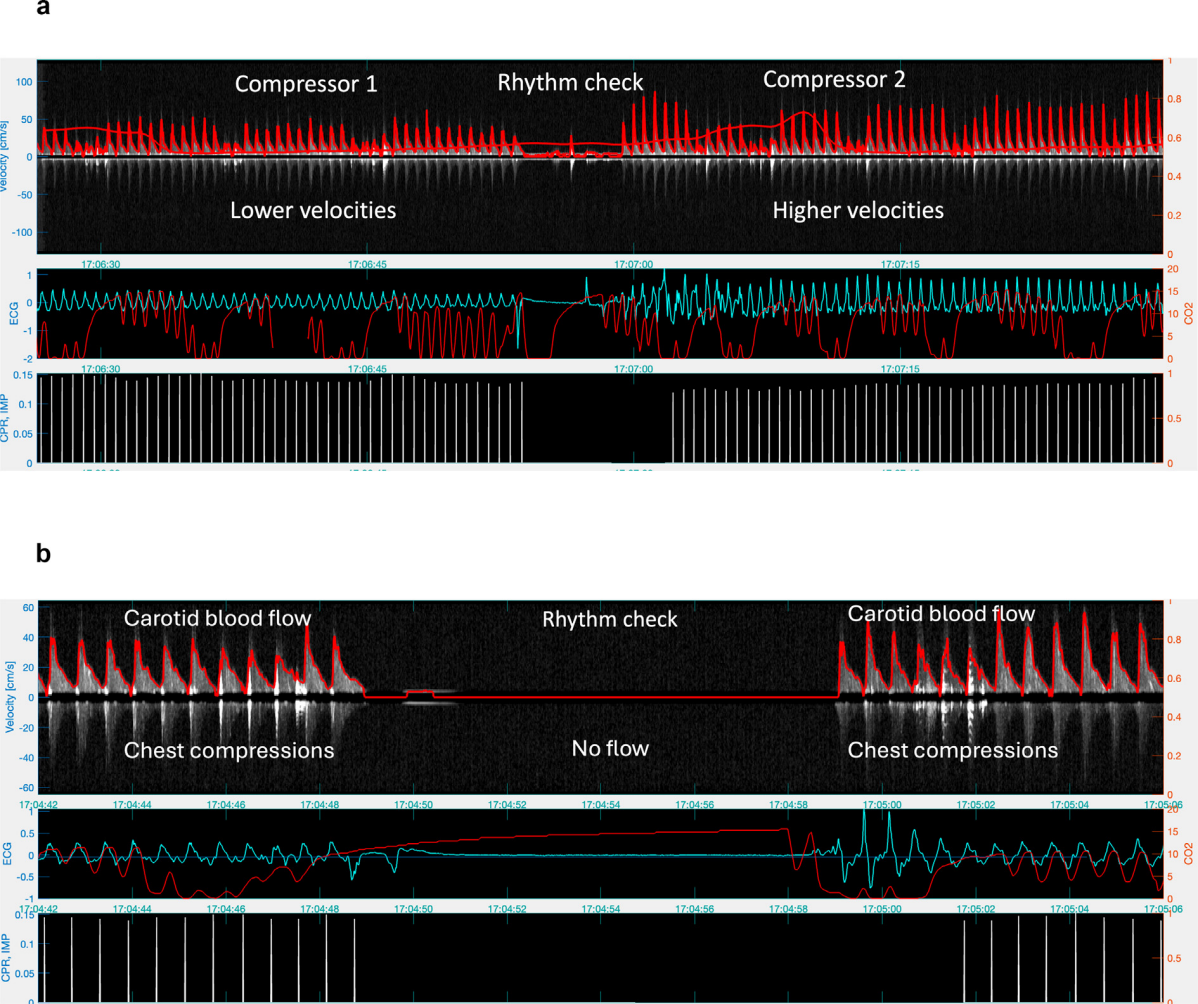
(a) Variation in peak carotid blood flow velocity generated by chest compressions by two different compressors. The upper panel displays carotid blood flow velocity. The middle panel shows the electrocardiogram (ECG) in blue and end-tidal CO_2_ in red_._ The lower panel presents the chest compression signal recorded by an accelerometer (CPR signal). Positive velocities in the Doppler spectrum, displayed above the baseline, indicate blood flow directed towards the brain. (b) Absence of carotid blood flow at rhythm assessment during asystole. Upper panel carotid blood flow velocity, middle panel blue line electrocardiogram (ECG) and red line end-tidal CO_2_, both with oscillations from chest compressions_,_ lower panel chest compression accelerometer (i.e. CPR) signal. Positive velocities in the Doppler spectrum, displayed above the baseline, indicate blood flow directed towards the brain. (For interpretation of the references to color in this figure legend, the reader is referred to the web version of this article.)

RescueDoppler data detected spontaneous circulation during ongoing chest compressions in four patients (Fig. 3a) and correctly identified temporary ROSC in 18 of 19 patients (Fig. 3b). Doppler blood flow velocities caused by chest compressions were confirmed in 31 patients. We observed different blood flow patterns in patients resuscitated with mechanical compression compared to patients with manual compressions (Fig. 4). In one patient, two rescuers generated very different peak systolic blood flow velocities (Fig. 5a), despite identical compression depth recorded by the accelerometer. A no-flow state was observed during rhythm checks in 24 patients, representing asystole (Fig. 5b), true pulseless electrical activity (PEA), or ventricular tachycardia/ventricular fibrillation (VT/VF).

### RescueDoppler system safety

No reports indicated that the RescueDoppler patch or system negatively affected CPR quality. The monitoring group found no adverse events or severe device deficiencies. Study personnel reported no delays in treatment, transport, or other procedures due to the systeḿs use. Summary of the questionnaire and safety reporting in Supplementary material, Appendix 3.

## Discussion

This pilot study demonstrated that continuous monitoring of carotid blood flow during CPR and cardiac arrest using the RescueDoppler system is safe and clinically informative, although feasibility needs to be improved. When operative, this novel hands-free ultrasound pulsed-wave system successfully detected spontaneous circulation, ROSC and chest compression-generated carotid blood flow velocity.

Real-time hemodynamic monitoring may become an important future component of personalized resuscitation. A *Resuscitation* editorial highlighted benefits of an individualized approach, such as continuous rhythm analysis, optimized defibrillation timing, real-time feedback on chest compressions and hand position, and monitoring of perfusion and oxygenation in critical organs using flow parameters.^16^ It emphasized precise drug administration timing and minimal or no interruption to chest compressions. The novel RescueDoppler system could play a crucial role in advancing this approach. An objective measure of circulation allows for proper intervention in due time (e.g., changing chest compression site, increasing depth or rate), potentially differentiate between PEA and pseudo-PEA, and allow for immediate assessment of the effect of defibrillation or medication.

### RescueDoppler prototype feasibility

The RescueDoppler patch was correctly placed over the left carotid artery in most cases, but adherence to the skin was poor in more than half of the patients. Developing a patch that adheres well even on moist or sweaty skin was challenging, and the neck is known to be a difficult area for patch placement.^17^ While many studies discuss self-adhesive patches^18^, ^19^, ^20^ and wearable technology; to our knowledge no one has addressed these challenges in emergency settings.

Poor data quality were sometimes caused by poor contact between the hydrogel covering the ultrasound probe and the skin. A more liquid gel could potentially improve skin contact and data quality. It is also very likely that unblinding the treating staff would improve the quality of the data since this would allow them to intervene in case of poor signal quality. Finally, once the device is in routine use it will not be forgotten by the resuscitation team.

Pulsed wave Doppler allows for active depth selection, thus enabling the RescueDoppler to differentiate between signals originating from veins and arteries. CPR-induced motion artifacts are present in all sample depths. However, by manually selecting the depth range of a vessel, RescueDoppler signals can be effectively identified. We also found that motion artifact velocities were much lower than typical blood velocities, meaning they are easily distinguishable small artifacts around the baseline of the Doppler spectrum. No other wearable ultrasound equipment does, to our knowledge, use this technology or demonstrates similar robustness.^21^, ^22^, ^23^

### RescueDoppler blood flow data

This pilot study confirms that blood flow signals were visible in most patients where the RescueDoppler patch adhered well. The RescueDoppler ultrasound probe features two angled elements to optimize skin contact and enhance blood flow measurements. The length of these elements that covers the carotid artery, combined with the application guide, contributed to these encouraging results. Despite the unknown insonation angle in this pilot study, the interpretation of blood flow patterns and velocity trends remain valuable. Furthermore, for the primary objectives — distinguishing true PEA from pseudo-PEA, identifying the optimal site for chest compressions, and detecting ROSC or asystole — the technology offers meaningful clinical insights.

CPR guidelines emphasize the importance of high-quality chest compressions,^1^, ^24^ with rhythm or pulse checks being the most common interruptions.^25^, ^26^ In this pilot study, the RescueDoppler identified spontaneous blood flow during ongoing chest compressions and rhythm checks, where the resuscitation team considered the patient to have PEA. This suggests that the RescueDoppler has a high sensitivity to detect blood flow long before a pulse can be palpated. These findings are supported by several studies.^6^, ^12^, ^27^

In some patients, the RescueDoppler detected variable peak blood flow velocities depending on the rescuer and the use of mechanical compression devices. The importance of hand positioning has been highlighted in various studies,^28^, ^29^, ^30^ although none have reported improved outcomes. The 2020 guidelines advise the use of physiological feedback to optimize hand positioning in individual patients as a knowledge gap.^3^

Different blood flow generated by different rescuers was observed in one patient. Factors such as age, sex and body composition influence anatomy, affecting optimal hand placement for chest compressions.^31^, ^32^, ^33^ Studies on physiologic feedback during CPR, such as invasive blood pressure and coronary perfusion have found an association with ROSC.^34^, ^35^, ^36^ These findings support personalized CPR, although these measurements are difficult to obtain during ongoing CPR. To the best of our knowledge, our findings are the first in humans to suggest that a Doppler ultrasound system could provide continuous feedback on the blood flow generated by different chest compressions and possibly different hand positioning.^13^

In half of the patients analyzed in this study, the RescueDoppler identified temporary ROSC with both systolic and diastolic blood flow. The device also detected spontaneous carotid blood flow that was not recognized by the resuscitation team, even during chest compressions. PEA is increasingly prevalent in cardiac arrest and studies have shown that both echocardiography and Doppler ultrasonography may alter management.^37^, ^38^ However, guidelines emphasize the need for skilled operators to minimize harm.^1^ Prolonged and frequent interruptions of chest compressions for rhythm checks are associated with adverse outcomes.^39^, ^40^, ^41^ Echocardiography has been shown to lengthen the duration of pulse checks, nearly doubling the recommended maximum of 10 s.^1^, ^25^ RescueDoppler successfully detected pseudo-PEA, as described in animal studies,^14^, ^27^ but further research is needed to support changes in the treatment protocols.

Venous signals were also visible in a majority of the RescueDoppler data when the patient had obtained ROSC. A few studies have used an increase in venous pressure as a potentially useful predictor of ROSC.^42^

Further clinical investigation into the RescueDoppler system is warranted. It could serve as pulse check tool, provide real-time feedback on hand positioning and chest compression quality, guide individual medication dosing, and potentially shortening rhythm checks. Additionally, unblinding and displaying blood flow data may complement ECG and EtCO_2_ monitoring, serving as a valuable adjunct in clinical assessment.

### RescueDoppler safety

The use of the RescueDoppler system and the patch by study personnel did not affect CPR quality, as reported in questionnaires and interviews. This finding aligns with another study on CPR interventions.^43^ Despite some device deficiencies, none were reported as severe or harmful. Notably, no thermal or mechanical damage were reported. The RescueDoppler system was confirmed safe for use.

### Limitations

This study noted several limitations associated with the use of a prototype device in the challenging environment of cardiac arrest emergencies. Frequent technical difficulties arose with the self-adhesive patch, requiring multiple modifications.

The RescueDoppler captures blood flow data from only one of the main arteries supplying the head and brain. In patients with left sided carotid artery disease, blood flow velocities may differ from those observed in individuals with healthy vasculature. To account for this, data on prior medical conditions were retrieved from the patients’ medical records following inclusion. Notably, none of the patients included in the analysis had a documented history of carotid artery disease.

Additionally, the demanding conditions inherent to emergency settings led to occasional equipment damage and incomplete data collection. Future development of the RescueDoppler systems will aim to enhance durability. In future studies the rescuer will be provided with unblinded real-time blood flow data. Another limitation was the absence of comparative data from established physiological monitoring methods. Although this limitation may be unavoidable given the study's context, future research should prioritize validating RescueDoppler-derived blood flow measurements against established techniques such as invasive blood pressure monitoring and/or EtCO_2_ measurements.

## Conclusion

Continuous hemodynamic monitoring of the common carotid artery during cardiac arrest and CPR is feasible with the novel RescueDoppler system. While technical challenges were encountered in this pilot study, the system proved to be easy to use, safe and did not compromise the quality of resuscitation. The RescueDoppler successfully detected spontaneous circulation during chest compressions, identified ROSC, and captured variations in blood flow velocities. This hands-free device could enable real-time personalized CPR, potentially transform cardiac arrest management and improving patient outcomes.

## Declaration of generative AI and AI-assisted technologies in the writing process

During the preparation of this work the author used Microsoft Copilot in selected and small parts of the first draft of the manuscript to improve language and to translate the questionnaire in the supplementary. After using this tool/service, the author reviewed and edited the content as needed and takes full responsibility for the content of the publication.

## Data availability

Data will be made available on request.

## CRediT authorship contribution statement

**Guro Mæhlum Krüger:** Writing – original draft, Visualization, Validation, Software, Project administration, Investigation, Formal analysis, Data curation. **Sunniva Gjerald Birkeli:** Writing – review & editing, Project administration, Investigation, Data curation. **Øystein Myrlund Hansen:** Visualization, Investigation, Data curation. **Bjørn Ove Faldaas:** Writing – review & editing, Conceptualization. **Anders Norvik:** Writing – review & editing, Investigation. **Hedda Juni Lund:** Writing – review & editing, Project administration. **Gregory Louis Egil Hautois:** Writing – review & editing, Investigation, Data curation. **John Helge Flage:** Investigation, Data curation. **Jon Urteaga:** Data curation. **Torbjørn Hergum:** Writing – review & editing, Visualization, Validation, Software, Conceptualization. **Hans Torp:** Visualization, Validation, Software, Formal analysis, Conceptualization. **Eirik Skogvoll:** Writing – review & editing, Supervision, Methodology. **Charlotte Björk Ingul:** Writing – review & editing, Validation, Supervision, Software, Resources, Project administration, Methodology, Investigation, Formal analysis, Data curation, Conceptualization.

## Funding

The RescueDoppler project has received funding from the Norwegian Research Council and from the Norwegian Health Association.

## Declaration of competing interest

The authors declare the following financial interests/personal relationships which may be considered as potential competing interests: “Charlotte B. Ingul reports financial support was provided by Norwegian Health Association. Charlotte B. Ingul reports financial support was provided by Norwegian Research Council. Charlotte B. Ingul reports financial support was provided by Cimon Medical. Hans Torp reports financial support was provided by Cimon Medical. Torbjoern Hergum reports financial support was provided by Cimon Medical. Charlotte B. Ingul reports a relationship with Cimon Medical that includes: consulting or advisory. Hans Torp reports a relationship with Cimon Medical that includes: employment and equity or stocks. Torbjoern Hergum reports a relationship with Cimon Medical that includes: employment and equity or stocks. Oistein Myrlund Hansen reports a relationship with GE Vingmed Ultrasound AS that includes: consulting or advisory. Hans Torp, Torbjoern Hergum has patent licensed to International (PCT) Patent Application No. PCT/EP2024/058678. If there are other authors, they declare that they have no known competing financial interests or personal relationships that could have appeared to influence the work reported in this paper”.
