# Supplementary material for: Realtime detection of spontaneous circulation in humans during cardiopulmonary resuscitation using a continuous hands-free carotid Doppler: a pilot study
Source: Resusc Plus. 2025 Sep 3;26:101080. doi: 10.1016/j.resplu.2025.101080 (PMC12492284; doi:10.1016/j.resplu.2025.101080)
Supplement: Supplementary Data 1 [file mmc1.docx]

# **SUPPLEMENTARY MATERIAL**

## **appendix 1**

## **methods**

### the rescuedoppler prototype patch

The self-adhesive patch was under continuous development during the study period. First the patch was designed with a thick foam material (supplier Nile AB, Ängelholm, Sweden) and the ultrasound transducer was coated with a thin hydrogel patch (supplier Axelgaard) to eliminate air gaps, enhance sound wave transmission and to reduce signal loss and artifacts. The foam material did not meet the extreme requirements of resuscitation and detached from the skin too easily. Inspired by wound care equipment utilized in the pre-hospital setting, the second patch consisted of hydrogel (Lohmann Nordic AB, Kungälv, Sweden), also with inconsistent results. During this phase, study personnel assembled patch components. Due to temporarily shortage of medical equipment, we used adhesive tape commonly employed in daily clinical practice to attach the probes. Further investigation and work lead to the patch with TPU (Thermoplastic Polyurethane) material supplied by Leonard Lang GmbH (Innsbruck, Austria), which also supplies the hydrogel coating for the ultrasound transducer and the final probe set up.

The application guide was developed by NTNU, Cimon and Inventas, Trondheim to make the placement of the RescueDoppler probe independent of the user. The application guide is patented (International (PCT) Patent Application No. PCT/EP2024/058678 Priority date: 30.03.2023).

### the rescuedoppler host unit

Several adjustments were made on the RescueDoppler system during the pilot study. The ultrasound engineers improved the software, enhanced the storage modality, and made the scanner-computer host unit more robust and user-friendly. Additionally, they worked on the cables and connectors between the probe and the host unit to secure data. Further improvements are not a focus aera, as we hope to unblind the RescueDoppler data in future investigations.

## **appendix 2**

**The RescueDoppler questionnaire**

**Evaluation Form for the Use of RescueDoppler Ultrasound System during Cardiac Arrest**

How much do you agree or disagree with the following statements (check one box) use free text if there are aspects you wish to highlight:

*Was RescueDoppler used during resuscitaion:*

| No  (describe why not in the free textfield) | Yes |
| --- | --- |
|  |  |

Free text:

***The instructions on the placement guide were easy to understand and provided enough information****:*

| Strongly agree | Partially agree | Neutral | Partially disagree | Strongly disagree |
| --- | --- | --- | --- | --- |
|  |  |  |  |  |

Free text:

***The patient's neck anatomy was favourable for placing the patch:***

| Strongly agree | Partially agree | Neutral | Partially disagree | Strongly disagree |
| --- | --- | --- | --- | --- |
|  |  |  |  |  |

Free text:

***It was easy to place the patch based on the illustration on the placement guide:***

| Strongly agree | Partially agree | Neutral | Partially disagree | Strongly disagree |
| --- | --- | --- | --- | --- |
|  |  |  |  |  |

Free text:

***It was easy to remove the first protective film from the patch:***

| Strongly agree | Partially agree | Neutral | Partially disagree | Strongly disagree |
| --- | --- | --- | --- | --- |
|  |  |  |  |  |

Free text:

***It was easy to remove the final protective film from the patch:***

| Strongly agree | Partially agree | Neutral | Partially disagree | Strongly disagree |
| --- | --- | --- | --- | --- |
|  |  |  |  |  |

Free text:

***I had received enough training in the use of the RescueDoppler ultrasound patch:***

| Strongly agree | Partially agree | Neutral | Partially disagree | Strongly disagree |
| --- | --- | --- | --- | --- |
|  |  |  |  |  |

Free text:

***The RescueDoppler ultrasound patch was not in the way during resuscitation****:*

| Strongly agree | Partially agree | Neutral | Partially disagree | Strongly disagree |
| --- | --- | --- | --- | --- |
|  |  |  |  |  |

Free text:

***Did any unwanted incidents occur related to wires and the RescueDoppler ultrasound patch****:*

| No | Yes (describe in the free text field) |
| --- | --- |
|  |  |

Free text:

***Did the placement of the RescueDoppler ultrasound patch need to be changed during the process****:*

| No | Yes (describe in the free text field) |
| --- | --- |
|  |  |

Free text:

***Was the RescueDoppler unit turned on right before the ultrasound patch was applied:***

| No | Yes |
| --- | --- |
|  |  |

Free text:

***Was the RescueDoppler unit turned on after the ultrasound patch was applied:***

| No | Yes |
| --- | --- |
|  |  |

Free text:

***Did the placement of the RescueDoppler ultrasound patch take time away from CPR****:*

| No | Yes (describe in the free text field) |
| --- | --- |
|  |  |

Free text:

***Was there any reaction on the neck due to the RescueDoppler ultrasound patch****:*

| No | Yes (describe in the free text field) |
| --- | --- |
|  |  |

Free text:

***How many were present during resuscitation****:*

| *Number:* |
| --- |
|  |

*Describe the affiliation and profession of those present:*

*Were there any other challenges not mentioned above that should be noted:*

| No | Yes (describe in the free text field) |
| --- | --- |
|  |  |

Free text:

*Questionnaire translated using Microsoft Copilot.*

## **appendix 3**

### rescuedoppler questionnaire and safety reporting

| **RescueDoppler use reported by study personnel** | Agree | Partly agree | Neutral | Partly disagree | Disagree | Missing | Total |
| --- | --- | --- | --- | --- | --- | --- | --- |
| The application aid was useful | 46 | 7 | 3 | . | 1 | 5 | 62 |
| I had sufficient training | 38 | 16 | 2 | . | 2 | 4 | 62 |
| The patch adhered well | 12 | 5 | 1 | 7 | 7 | 30 | 62 |
| The RD patch did not interfere with CPR | 48 | 4 | 3 | . | 2 | 5 | 62 |
|  | | | | | | | |
| **RescueDoppler reported safety by study personnel** | Yes | | No | | Missing | | Total |
| Were there any challenges due to the RD system | 13 | | 44 | | 5 | | 62 |
| Were there any skin reactions | 2 | | 53 | | 7 | | 62 |
|  | | | | | | | |
| **RescueDoppler safety**  **(Clinical monitoring committee)** | Yes | | | No | | | |
| Adverse Events | 0 | | | 62 | | | |
| Protocol deviation | | | | | | | |
| Inclusion/Exclusion | 2 | | | 60 | | | |
| Device deficiency |  | | |  | | | |
| RD cable | 10 | | | 52 | | | |
| RD box | 10 | | | 52 | | | |
| RD patch detached | 15 | | | 47 | | | |
| Did the deviation have significance for the  patient? | 0 | | | 62 | | | |

*Appendix 3. Summary of the questionnaire and safety reporting by the clinical monitoring committee. The questionnaire was completed by the study personnel following each cardiac arrest response. Some of the questions were initially omitted, but were subsequently followed up by the study group, including the question regarding the patch adhesion. Reported challenges by the study personnel were among others: moisty, hairy skin; strain to the cables-> patch displacement; the box failed to power on; the liner was not removed, affecting proper adhesion. There were no protocol deviations or device deficiencies of importance for the patients. RD= RescueDoppler. CPR= Cardiopulmonary resuscitation.*
